# The Fecal Microbiome in Dogs with Acute Diarrhea and Idiopathic Inflammatory Bowel Disease

**DOI:** 10.1371/journal.pone.0051907

**Published:** 2012-12-26

**Authors:** Jan S. Suchodolski, Melissa E. Markel, Jose F. Garcia-Mazcorro, Stefan Unterer, Romy M. Heilmann, Scot E. Dowd, Priyanka Kachroo, Ivan Ivanov, Yasushi Minamoto, Enricka M. Dillman, Jörg M. Steiner, Audrey K. Cook, Linda Toresson

**Affiliations:** 1 Gastrointestinal Laboratory, Small Animal Clinical Sciences, College of Veterinary Medicine and Biomedical Sciences, Texas A&M University, College Station, Texas, United States of America; 2 Facultad de Medicina Veterinaria, Universidad Autónoma de Nuevo León. Gral. Escobedo, Nuevo León, México; 3 Clinic of Small Animal Medicine, Ludwig-Maximillians-University, Munich, Germany; 4 Molecular Research DNA Laboratory, Shallowater, Texas, United States of America; 5 College of Veterinary Medicine and Biomedical Sciences, Texas A&M University, College Station, Texas, United States of America; 6 Helsingborg Referral Animal Hospital, Helsingborg, Sweden; Charité, Campus Benjamin Franklin, Germany

## Abstract

**Background:**

Recent molecular studies have revealed a highly complex bacterial assembly in the canine intestinal tract. There is mounting evidence that microbes play an important role in the pathogenesis of acute and chronic enteropathies of dogs, including idiopathic inflammatory bowel disease (IBD). The aim of this study was to characterize the bacterial microbiota in dogs with various gastrointestinal disorders.

**Methodology/Principal Findings:**

Fecal samples from healthy dogs (n = 32), dogs with acute non-hemorrhagic diarrhea (NHD; n = 12), dogs with acute hemorrhagic diarrhea (AHD; n = 13), and dogs with active (n = 9) and therapeutically controlled idiopathic IBD (n = 10) were analyzed by 454-pyrosequencing of the 16S rRNA gene and qPCR assays. Dogs with acute diarrhea, especially those with AHD, had the most profound alterations in their microbiome, as significant separations were observed on PCoA plots of unweighted Unifrac distances. Dogs with AHD had significant decreases in Blautia, Ruminococcaceae including *Faecalibacterium*, and *Turicibacter* spp., and significant increases in genus *Sutterella* and *Clostridium perfringens* when compared to healthy dogs. No significant separation on PCoA plots was observed for the dogs with IBD. *Faecalibacterium* spp. and Fusobacteria were, however, decreased in the dogs with clinically active IBD, but increased during time periods of clinically insignificant IBD, as defined by a clinical IBD activity index (CIBDAI).

**Conclusions:**

Results of this study revealed a bacterial dysbiosis in fecal samples of dogs with various GI disorders. The observed changes in the microbiome differed between acute and chronic disease states. The bacterial groups that were commonly decreased during diarrhea are considered to be important short-chain fatty acid producers and may be important for canine intestinal health. Future studies should correlate these observed phylogenetic differences with functional changes in the intestinal microbiome of dogs with defined disease phenotypes.

## Introduction

Recent molecular-phylogenetic studies have revealed a complex assembly of bacteria in the mammalian gastrointestinal (GI) tract [1]–[3]. Intestinal microbes play a crucial role in the maintenance of host health. They act as a defending barrier against transient pathogens, support the host in digestion and energy harvest from the diet, stimulate the immune system, and provide nutritional support for enterocytes [4].

The intestinal microbiota has also been implicated in the pathogenesis of various canine GI disorders, either associated with the presence of specific pathogens (e.g., enterotoxigenic *C. perfringens*, *Salmonella*, viruses, and parasites) in acute episodes of diarrhea [5], [6], or a non-specific dysbiosis such as that described in dogs with idiopathic inflammatory bowel disease [7]–[10]. Canine idiopathic IBD is one of the most common causes of chronic GI disease in dogs and encompasses a group of chronic enteropathies of unknown cause, which are characterized by infiltration of the intestinal mucosa with inflammatory cells [11]. Although histopathologic changes may be found in any segment of the GI tract, the small intestine is typically the most frequently affected segment. The diagnosis of idiopathic IBD is made after known causes of GI inflammation have been ruled out, the animal has not shown a favorable response to a dietary and antibiotic therapeutic trial, and typically requires immunosuppressive or anti-inflammatory therapy [11].

Molecular-phylogenetic studies have revealed a bacterial and/or fungal dysbiosis in the duodenum of dogs with idiopathic IBD. Most commonly, a decrease in the proportions of Clostridiales and an increase in Proteobacteria is observed [7], [9], [10], [12]. Only few molecular studies have described the fecal microbiota of dogs with acute and chronic GI disorders. One study, using fluorescent *in situ* hybridization (FISH) probes, found *Bacteroides* counts to be significantly increased in Beagle dogs with chronic diarrhea [13]. In contrast, using 454-pyrosequencing of the cpn60 gene, significantly decreased proportions of Bacteroidetes were observed in dogs with unspecified diarrhea [14]. Using terminal restriction fragment length polymorphism (T-RFLP) analysis and quantitative PCR (qPCR), an increased abundance of *Clostridium perfringens*, *Enterococcus faecalis*, and *E. faecium* was observed in dogs during diarrheic episodes [15]. While these studies suggest a dysbiosis present in fecal samples of dogs with diarrhea, additional studies using high-throughput sequencing technologies in dogs with well-defined acute and chronic disease phenotypes are needed to further characterize changes in the fecal microbiome. In addition, comparison of fecal findings in dogs with IBD with those previously observed in duodenal biopsies is of interest [7], as collection of fecal samples is more practical. Furthermore, it is unclear if the pattern of dysbiosis observed in dogs with IBD is specific for this disorder, or if similar patterns are present in acute GI diseases.

This study compared the fecal microbiome of healthy dogs, dogs with acute non-hemorrhagic diarrhea (NHD), dogs with acute hemorrhagic diarrhea (AHD), and dogs with active and therapeutically controlled clinically insignificant IBD. The results indicate differences in the fecal microbiome among the dogs with various GI diseases. Dogs with acute diarrhea had the most pronounced changes, with several bacterial groups altered when compared to healthy dogs. Only *Faecalibacterium* spp. and Fusobacteria were decreased in dogs with clinically active IBD, but increased during time periods of clinically insignificant IBD.

## Materials and Methods

### Ethics Statement

The collection and analysis of fecal samples was approved by the institutional Clinical Research Review Committee of the College of Veterinary Medicine, Texas A&M University (CRRC#09-06).

### Animals and Sample Collection

Fecal samples from a total of 76 dogs were analyzed. These dogs were either healthy (n = 32), or had signs of either acute non-hemorrhagic diarrhea (NHD; n = 12), acute hemorrhagic diarrhea (AHD, n = 13), active inflammatory bowel disease (A-IBD; n = 9), or therapeutically controlled clinically insignificant IBD (S-IBD; n = 10), as scored by a published canine clinical IBD activity index (CIBDAI) [16]. Left-over naturally-passed feces collected for routine fecal examination were frozen within a few hours of collection at either −20°C or −80°C, and were stored frozen until processing of samples for DNA extraction. The summary of the baseline characteristics for each animal group is listed in Table 1, and detailed descriptions of each enrolled dog are listed in supplementary Tables S1–S3.

**Table 1:** Summary of basic characteristics and alpha diversity measures.

	Healthy	NHD	AHD	A_IBD	S_IBD	p-value
Age (years; median, range)	4.6, 0.3–15.0	5.3, 0.5–15.0	5.0, 2.0–16.0	3.5, 0.6–7.6	5.7, 3.7–8.7	0.496
Weight (lbs; median, range)	47.0, 5.8–81.5	47.1, 5.5–75.0	19.8, 4.0–68.3	55.0, 9.0–130.0	56.1, 18.5–91.7	0.574
CIBDAI (median, range)	N/A	N/A	N/A	7, 5–9	1.5, 1–2	<0.001
gender (female/male)	14/18	8/4	7/6	3/5	3/7	0.449
Country	Sweden n = 8; USA n = 24	USA	Germany	Sweden	Sweden	n/a
OTU_97_ (mean ± SD)	242±93	188±95	175±57	163±91	119±85	0.111
Shannon index (mean ± SD)	3.3±0.8	2.4±1.4	2.6±0.9	2.3±1.1	1.9±1.0	0.104
Chao1 (mean ± SD)	504±181	390±232	327±126	357±212	251±203	0.053

IBD = inflammatory bowel disease.

CIBDAI = canine IBD disease activity index.

NHD = acute non-hemorrhagic diarrhea, AHD = acute hemorrhagic diarrhea, A_IBD = active IBD, S_IBD = clinically insignificant IBD.

#### Healthy controls

Fecal samples from a total of 32 pet dogs were analyzed by 454-pyrosequencing and quantitative PCR assays (qPCR). All dogs were privately owned and lived in diverse home environments, were on a variety of commercial diets, and none of the dogs had a history of gastrointestinal signs or administration of antibiotics for at least the past 3 months (Table S1). Eight healthy dogs lived in Sweden, while the remaining 24 healthy dogs lived in Texas, USA.

#### Dogs with acute non-hemorrhagic diarrhea (NHD)

Fecal samples from a total of 12 pet dogs that presented to a first-opinion practice (Austin, TX) with acute, uncomplicated, non-hemorrhagic diarrhea were evaluated (duration of diarrhea <3 days). Of those, 7 samples were analyzed by 454-pyrosequencing, while all 12 samples were analyzed by qPCR assays. None of the dogs had a previous history of GI signs or had received antibiotics within the previous 3 months (Table S2). Diagnostic evaluation included complete blood count (CBC), serum chemistry profiles, and partial fecal analysis for enteric pathogens by fecal flotation and fecal cytology. *Clostridium perfringens* enterotoxin and *C. difficile* toxin A/B were analyzed using commercially available ELISA kits (*C. perfringens* Enterotoxin Test™ and *C. difficile* Tox A/B II™, TechLab, Blacksburg, VA). Based on review of the medical records, all dogs in this group recovered after non-specific symptomatic therapy (e.g., fluid supplementation, gastric acid blockers) within a few days.

#### Dogs with acute hemorrhagic diarrhea (AHD)

Fecal samples were analyzed from a total of 13 pet dogs that presented to the Clinic of Small Animal Medicine, LMU University of Munich, Germany, with acute hemorrhagic diarrhea (duration of <3 days). None of the dogs had a previous history of GI signs or had received antibiotics within the previous 3 months (Table S2). Diagnostic evaluation included CBC, serum chemistry profiles, and partial fecal analysis for enteric pathogens (*C. perfringens* enterotoxin ELISA, *C. difficile* toxin A/B ELISA, and fecal culture).

#### Dogs with idiopathic IBD

Fecal samples were analyzed from pet dogs that had been presented to the Helsingborg Referral Animal Hospital, Helsingborg, Sweden with signs of chronic GI disease. Dogs underwent clinical evaluation by a veterinary internist (LT). Diagnostic tests that were performed included a CBC, serum chemistry profiles, fecal flotation, serum concentrations of cobalamin and folate, and depending on the clinical signs, serum concentrations of trypsin-like immunoreactivity (cTLI) and pancreatic lipase-immunoreactivity (cPLI). During the months of diagnostic work-ups, dogs underwent various forms of antibiotic and/or dietary trials. All dogs failed the trials and subsequently underwent endoscopy with collection of intestinal biopsies. All dogs then received and responded to immunosuppressive therapy, leading to a diagnosis of idiopathic IBD (Table S3).

The disease activity of these dogs was scored using the published clinical canine IBD activity index (CIBDAI) [16]. The CIBDAI is based on 6 criteria, each scored on a scale from 0–3: attitude/activity, appetite, vomiting, stool consistency, stool frequency, and weight loss. The total composite score is determined to be clinically insignificant (score 0–3), mild (score 4–5), moderate (score 6–8), or severe (score 9 or greater). We analyzed a total of 19 dogs (Table S3). Of those 19 dogs, 9 were newly diagnosed with active IBD (A_IBD) as judged by their CIBDAI score (median 7, range 5–9), and fecal samples collected at the time of diagnosis were analyzed (5 samples were analyzed by pyrosequencing; all 9 samples were analyzed by qPCR). The other 10 dogs had been on medical treatment (Table S3) for their idiopathic IBD for several months to years (therapeutically controlled stable IBD; S_IBD) and had clinically insignificant or no signs of IBD as scored by the CIBDAI (median 1.5, range 1–2) at the time of sample collection. From the latter group of dogs all 10 samples were analyzed by pyrosequencing and qPCR assays. None of these 19 dogs received antibiotics for at least 2 months before sample collection.

In addition, paired samples were obtained from 8 dogs, representing time points when, based on CIBDAI scoring, the dogs showed either a clinically significant CIBDAI (median 5, range 4–9) or a clinically insignificant CIBDAI (median 1.5, range 1–3). The time period between the collections of repeated samples ranged from 2–8 months (median 5.5 months). These paired samples were analyzed separately by qPCR assays, as these follow up samples were obtained after the pyrosequencing analysis had been completed.

### DNA Extraction

An aliquot of 100 mg (wet weight) of each fecal sample was extracted by a bead-beating method using a commercial DNA extraction kit (ZR Fecal DNA Kit™, Zymo Research Corporation) following the manufacturer’s instructions. The bead beating step was performed on a homogenizer (FastPrep-24, MP Biomedicals) for 60 s at a speed of 4 m/s.

### 454-pyrosequencing

Bacterial tag-encoded FLX-titanium amplicon pyrosequencing (bTEFAP) based on the V1–V3 region (*E. coli* position 27–519) of the 16 S rRNA gene was performed on 67 of the 77 samples as described previously with primers forward 28F: GAGTTTGATCNTGGCTCAG and reverse 519R: GTNTTACNGCGGCKGCTG [3], [17]. Raw sequence data were screened, trimmed, denoised, and filtered with the QIIME pipeline version 1.4.0 (http://qiime.sourceforge.net) [18] with the following settings: minimum read length of 300 bp; no ambiguous base calls; no homopolymeric runs longer than 8 bp; average quality value>q25 within a sliding window of 50 bp. Chimeras were excluded using the software B2C2 (http://www.researchandtesting.com/B2C2.html) [7], [19]. Operational taxonomic units (OTUs) were defined as sequences with at least 97% similarity using QIIME. For classification of sequences on a genus level the naïve Bayesian classifier within the Ribosomal Database Project (RDP, v10.28) was used. The confidence threshold in RDP was set to 80% [7].

### Quantitative PCR (qPCR)

For validation of pyrosequencing results and/or to evaluate bacterial groups that are typically present at very low abundance or typically not detected in sequence data based on the authors’ experience from previous studies (i.e., *Bifidobacterium* spp.; *Faecalibacterium* spp.) [3], [20], [21], qPCR assays for selected bacterial groups were performed: total bacteria, Bacteroidetes, Fusobacteria, *Blautia*, Ruminococcaceae, *Faecalibacterium* spp., *Turicibacter* spp., *Bifidobacterium* spp., and *Clostridium perfringens*. PCR was also used to detect the genes encoding *C. perfringens* enterotoxin (*cpe*) and *C. difficile* toxin B (*tcd* B). Real-time PCR conditions were performed as described previously [22]. The oligonucleotide sequences of primers and probes, and respective annealing temperatures are summarized in Table 2. The qPCR data was expressed as log amount of DNA (fg) for each particular bacterial group per 10 ng of isolated total DNA.

**Table 2:** Oligonucleotides primers/probes used in this study.

qPCR primers/probe	Sequence (5′-3′)	Target	Annealing (°C)	Reference
CFB555f	CCGGAWTYATTGGGTTTAAAGGG	Bacteroidetes	60	[38]
CFB968r	GGTAAGGTTCCTCGCGTA			
BifF	TCGCGTCYGGTGTGAAAG	*Bifidobacterium*	60	[39]
BifR	CCACATCCAGCRTCCAC			
FaecaF	GAAGGCGGCCTACTGGGCAC	*Faecalibacterium*	60	[22]
FaecaR	GTGCAGGCGAGTTGCAGCCT			
RumiF	ACTGAGAGGTTGAACGGCCA	Family Ruminococcaceae	59	[22]
RumiR	CCTTTACACCCAGTAAWTCCGGA			
CPerf165F	CGCATAACGTTGAAAGATGG			
CPerf269R	CCTTGGTAGGCCGTTACCC	*C. perfringens* 16S	58	[40]
CPerf187F (probe)	TCATCATTCAACCAAAGGAGCAATCC			
TM-cpe-F	AACTATAGGAGAACAAAATACAATAG			
TM-cpe-R	TGCATAAACCTTATAATATACATATTC	*C. perfringens e*nterotoxin	55	[41]
TM-cpe-pr	TCTGTATCTACAACTGCTGGTCCA			
tcdB-F	GGTATTACCTAATGCTCCAAATAG			
tcdB-R	TTTGTGCCATCATTTTCTAAGC	*C. difficile* toxin B gene	58	[42]
tcdB-P (probe)	ACCTGGTGTCCATCCTGTTTCCCA			
Fuso-F	KGGGCTCAACMCMGTATTGCGT	Fusobacterium	51	This study
Fuso-R	TCGCGTTAGCTTGGGCGCTG			
Blaut-F	TCTGATGTGAAAGGCTGGGGCTTA	*Blautia spp.*	56	This study
Blaut-R	GGCTTAGCCACCCGACACCTA			
341-F	CCTACGGGAGGCAGCAGT	Universal Bacteria	59	[43]
518-R	ATTACCGCGGCTGCTGG			
TuriciF	CAGACGGGGACAACGATTGGA	*Turicibacter*	63	This study
TuricR	TACGCATCGTCGCCTTGGTA			

### Statistical Analysis

To account for unequal sequencing depth across samples, and to avoid exclusion of samples with lower number of sequence reads, the subsequent analysis was performed on a randomly selected subset of 2,000 sequences per sample. Differences in microbial communities between disease groups were investigated using the phylogeny-based unweighted UniFrac distance metric, and PCoA plots and rarefaction curves were plotted using QIIME. To determine differences in microbiota composition between the animal groups, the analysis of similarities (ANOSIM) function in the statistical software package PRIMER 6 (PRIMER-E Ltd., Lutton, UK) was used on the unweighted UniFrac distance matrixes. To visualize the relative abundance of bacterial families for individual fecal samples, heat maps were generated in NCSS 2007 (NCSS, Kaysville, Utah).

For all datasets, assumption of normality was tested using the D’Agostino and Pearson normality test (Prism 5.00, GraphPad Software Inc.). Only bacterial taxa that were present in at least 50% of dogs (either healthy or diseased) were included in the analysis. Because most datasets did not meet the assumptions of normal distribution, the differences in the proportions of bacterial taxa (defined as percentage of total sequences) or qPCR results between healthy and disease groups were determined using non-parametric Kruskal-Wallis tests (Prism v5.00, GraphPad Software Inc.). The resulting p-values of the Kruskal-Wallis tests were corrected for multiple comparisons on each phylogenetic level using the Benjamini & Hochberg’s False Discovery Rate, and a *p*<0.05 was considered statistically significant [23]. For those bacterial groups that were still significant after p-value adjustment, a Dunns’ post-test was used to determine which disease categories were significantly different. A Fisher’s exact test was used to determine the proportions of dogs that harbored specific bacterial taxa or toxins.

Paired samples were available for 8 dogs with IBD, representing samples at time of clinically active and therapeutically controlled IBD (i.e., clinically insignificant IBD), respectively. The qPCR assays for these paired samples were performed as batch in the same assay run, and the obtained data for the paired time points were compared using a Wilcoxon signed rank test.

Linear Discriminant Analysis (LDA) was used to achieve dimensionality reduction and thereby to identify combinations of bacterial groups that would discriminate between healthy dogs and all diseased dogs (independent of disease phenotype) [24]. Using the OTUs as features for the classification, the single, two, or three feature LDA classifiers were constructed and ranked based on their error estimates [25], [26]. More detailed descriptions of LDA classification are provided as supplementary Method S1.

## Results

### Animals and Disease Characteristics

No significant differences in age, gender, or body weight were found among the various animal groups (Table 1).

### Sequence Analysis

**Figure 1:**
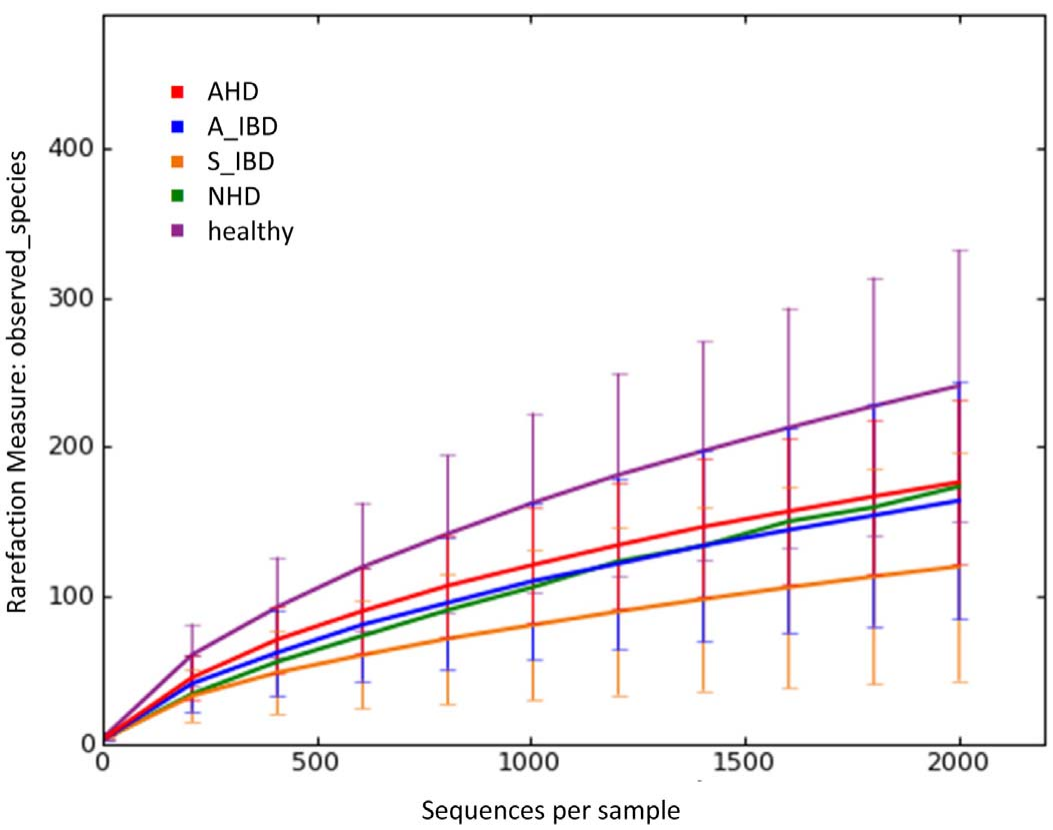
Rarefaction analysis of 16 S rRNA gene sequences obtained from canine fecal samples. Lines represent the average of each group, while the error bars represent the standard deviations. The analysis was performed on a randomly selected subset of 2,000 sequences per sample. A_IBD = active IBD; S_IBD = therapeutically controlled IBD; NHD = acute non-hemorrhagic diarrhea; AHD = acute hemorrhagic diarrhea.

The 454- pyrosequencing pipeline yielded 189,138 quality sequences for the 67 samples analyzed. Across all disease groups, sequences were classified into nine bacterial phyla (Table 3). Figure 1 illustrates the rarefaction curves for all disease groups. No significant differences in the number of OTUs, the Shannon index, and the Chao1 metric were observed (Table 1).

**Table 3:** Relative percentages of the most abundant bacterial groups at the various phylogenetic levels (phylum, class, order, family, genus) based on pyrosequencing.

	Medians % (min-max%)*					
	Healthy	NHD	AHD	Active IBD	Controlled IBD	Kruskal-WallisP-value**
**Firmicutes**	96.6 (81–100)	95.6 (83–100)	56 (13–100)	98.7 (95–100)	98.6 (24–100)	0.0985
Clostridia	78.1 (21–97)	86.8 (46–99)	55.6 (12–99)	45.5 (1–94)	47 1–91)	1
Clostridiales	78.1 (21–97)	86.8 (46–99)	55.6 (12–99)	45.5 (1–94)	47 (1–91)	1
Clostridiaceae	36.2 (6–84)	81.5 (32–99)	46.4 (7–99)	26.4 (1–82)	18.2 (1–75)	0.302
* Clostridium*	33.7^a^ (5–84)	81.5^b^ (32–99)	44.0^a,b^ (6–99)	13.7^a^ (0–82)	14.2^a^ (0–82)	**0.03**
Ruminococcaceae	16.0^a^ (0–46)	4.7^b^ (0–21)	0.8^b^ (0–18)	5.6^a,b^ (0–54)	7.9^a,b^ (0–53)	**0.004**
* Faecalibacterium*	0.1 (0–16)	0 (0–5)	0 (0–3)	0 (0–0)	0.3 (0–3)	1
* Ruminococcus*	15.4^a^ (0–46)	4.7^b^ (0–16)	0.7^b^ (0–18)	5.6^a,b^ (0–54)	6.8^a,b^ (0–53)	**0.008**
Lachnospiraceae	0.4 (0–2)	0.1 (0–3)	0 (0–1)	0.3 (0–1)	0.3 (0–3)	0.114
* Blautia*	9.9^a^ (0–28)	0.2^b^ (0–17)	0.2^b^ (0–4)	5.9^a,b^ (0–9)	3.6^b^ (0–16)	**0.002**
* Roseburia*	0.2 (0–1)	0.1 (0–3)	0 (0–1)	0.1 (0–0)	0.1 (0–1)	0.642
* Coprococcus*	0 (0–1)	0 (0–0)	0 (0–0)	0.1 (0–0)	0.1 (0–1)	1
Veillonellaceae	0 (0–4)	0 (0–0)	0 (0–1)	0 (0–0)	0 (0–0)	1
Eubacteriaceae	0.2 (0–2)	0.1 (0–13)	0.1 (0–17)	0.2 (0–0)	0.5 (0–4)	1
* Eubacterium*	0.8 (0–27)	0.1 (0–13)	0.1 (0–17)	0.3 (0–1)	1 (0–5)	0.564
Erysipelotrichi	7.8^a^ (0–45)	0.7^a,b^ (0–9)	0.1^b^ (0–2)	0.8^a,b^ (0–99)	0.8^b^ (0–8)	**0.0009**
Erysipelotrichales	7.8^a^ (0–45)	0.7^a,b^ (0–9)	0.1^b^ (0–2)	0.8^a,b^ (0–99)	0.8^b^ (0–8)	**0.0009**
Erysipelotrichaceae	7.8^a^ (0–45)	0.7^a,b^ (0–9)	0.1^b^ (0–2)	0.8^a,b^ (0–99)	0.8^b^ (0–8)	**0.0009**
* Turicibacter*	0.5 (0–39)	0.1 (0–4)	0 (0–0)	0 (0–0)	0.1 (0–1)	0.138
* Allobaculum*	0.3 (0–14)	0.4 (0–8)	0 (0–1)	0 (0–3)	0 (0–2)	1
Bacilli	0 (0–15)	0.2 (0–16)	0 (0–3)	0 (0–1)	0 (0–1)	0.2169
Lactobacillales	0.2 (0–74)	1.7 (0–29)	0.4 (0–5)	18.2 (0–60)	7.9 (0–98)	1
Streptococcaceae	0.1 (0–74)	0.3 (0–19)	0.1 (0–4)	6 (0–60)	2.7 (0–95)	1
* Streptococcus*	0 (0–74)	0.3 (0–19)	0.1 (0–4)	3.4 (0–60)	2.5 (0–95)	1
Lactobacillaceae	0 (0–61)	0 (0–2)	0 (0–0)	0 (0–11)	0.2 (0–98)	0.86
Enterococcaceae	0 (0–3)	0 (0–2)	0 (0–3)	0 (0–1)	0 (0–1)	1
**Proteobacteria**	0.30^a^ (0–3)	1.3^a,b^ (0–16)	4.3^b^ (0–17)	0.1^a^ (0–1)	0.1^a^ (0–46)	**0.016**
Betaproteobacteria	0.0^a^ (0–0)	0.0^a,b^ (0–3)	2.1^b^ (0–14)	0.0^a,b^ (0–0)	0.0^a^ (0–8)	**0.0099**
* Sutterella*	0.0^a^ (0–0)	0.0^a^ (0–0)	1.6^b^ (0–14)	0.0^a^ (0–0)	0.0^a^ (0–1)	**0.008**
Gammaproteobacteria	0 (0–3)	1 (0–16)	0.6 (0–15)	0 (0–0)	0 (0–29)	0.0648
Enterobacteriales	0 (0–3)	0.2 (0–16)	0.1 (0–13)	0 (0–0)	0 (0–19)	1
Enterobacteriaceae	0 (0–0)	0.2 (0–16)	0.1 (0–13)	0 (0–0)	0 (0–19)	1
Alphaproteobacteria	0.1 (0–0.9)	0.1 (0–1.2)	0 (0–0.2)	0.1 (0–0.3)	0 (0–2)	0.063
Rickettsiales	0.1^a^ (0–0.9)	0.0^a,b^ (0–0.2)	0.0^b^ (0–0.2)	0.1^a,b^ (0–0.3)	0.0^b^ (0–0.4)	**0.0072**
Anaplasmataceae	0.1^a^ (0–0.9)	0.0^a,b^ (0–0.2)	0.0^b^ (0–0.2)	0.1^a,b^ (0–0.3)	0.0^b^ (0–0.4)	**0.016**
* Anaplasma*	0.1^a^ (0–0.9)	0.0^a,b^ (0–0.2)	0.0^b^ (0–0.2)	0.1^a,b^ (0–0.3)	0.0^b^ (0–0.4)	**0.016**
**Bacteroidetes**	0 (0–18)	0 (0–3)	0.5 (0–17)	0 (0–0)	0 (0–12)	0.0685
Bacteroidia	0 (0–18)	0 (0–0)	0.5 (0–17)	0 (0–0)	0 (0–11)	0.1683
Bacteroidales	0 (0–18)	0 (0–0)	0.5 (0–17)	0 (0–0)	0 (0–11)	0.1683
Bacteroidaceae	0 (0–4)	0 (0–0)	0.5 (0–10)	0 (0–0)	0 (0–3)	0.524
* Bacteroides*	0 (0–3)	0 (0–0)	0.5 (0–10)	0 (0–0)	0 (0–3)	0.696
**Actinobacteria**	1.8^a^ (0–13)	1.4^a.b^ (0–6)	0.2^b^ (0–3)	0.8^a,b^ (0–5)	1.0^a,b^ (0–15)	**0.019**
Actinobacteria (class)	1.8^a^ (0–13)	1.4^a.b^ (0–6)	0.2^b^ (0–3)	0.8^a,b^ (0–5)	1.0^a,b^ (0–15)	**0.0342**
Coriobacteriales	1.8^a^ (0–13)	1.0^a,b^ (0–6)	0.1^b^ (0–2)	0.8^a,b^ (0–5)	0.7^a,b^ (0–15)	**0.0162**
Coriobacteriaceae	1.8^a^ (0–13)	1.0^a,b^ (0–6)	0.1^b^ (0–2)	0.8^a,b^ (0–5)	0.7^a,b^ (0–15)	**0.036**
* Collinsella*	1.5^a^ (0–13)	1.0^a,b^ (0–4)	0.0^b^ (0–2)	0.7^a,b^ (0–5)	0.5^a,b^ (0–13)	**0.018**
**Fusobacteria**	0.1 (0–4)	0 (0–2)	23.5 (0–75)	0 (0–0)	0 (0–17)	0.0865
Fusobacteriales	0 (0–4)	0 (0–2)	23.4 (0–0)	0 (0–0)	0 (0–14)	0.1647
Fusobacteriaceae	0 (0–4)	0 (0–2)	23.4 (0–0)	0 (0–0)	0 (0–14)	0.366
* Fusobacterium*	0 (0–4)	0 (0–2)	23.2 (0–0)	0 (0–0)	0 (0–6)	0.5

Taxa present in at least 50% of dogs (either healthy or diseased) included in analysis.

** p-values adjusted based on the Benjamini and Hochberg False discovery rate.

* Medians not sharing a common superscript are significantly different (p<0.05 based on a Dunn’s multiple comparisons test).

NHD = acute hemorrhagic diarrhea; AHD = acute hemorrhagic diarrhea; IBD = inflammatory bowel disease.

### Microbial Communities in Controls and in Dogs with Acute and Chronic Gastrointestinal Diseases

No significant differences were observed in total bacterial abundance among the groups based on qPCR analysis (Table 4; p = 0.09). Significant differences in microbial communities were, however, observed among the various groups. According to the linear discriminant analysis (LDA; Method S1), the triple combination of *Blautia* spp., *Faecalibacterium* spp., and *Turicibacter* spp. had the highest discriminatory power when healthy dogs were compared to all the dogs with gastrointestinal disease (independent of disease phenotype).

**Table 4:** Summary statistics for qPCR results.

	Medians (min-max)* log DNA					
	Healthy	NHD	AHD	Active IBD	Controlled IBD	Kruskal-WallisP-value
Bacteroidetes	6.4^a^ (0.0–12.1)	4.5^b^ (0.0–6.1)	6.7^a^ (4.7–9.9)	7.3^a^ (0.0–9.3)	5.3^a,b^ (0.0–7.5)	0.0009
Bifidobacterium	2.9 (0.0–7.3)	3.1 (0.0–6.2)	0 (0.0–3.9)	2.2 (0.0–6.6)	3.8 (0.0–7.5)	0.0959
Blautia	9.7^a^ (8.2–10.7)	6.3^b^ (5.7–10.2)	8.2^b^ (6.9–10.2)	9.2^a,b^ (7.3–9.9)	9.5^a,b^ (5.9–9.9)	0.0003
C. perfringens	2.0^a^ (0.0–6.1)	4.0^a,b^ (0.0–7.4)	6.2^b^ (0.6–7.6)	3.0^a,b^ (0.0–6.7)	2.4^a,b^ (0.6–5.7)	0.0002
Faecalibacterium	5.8^a^ (4.1–7.9)	0.0^b^ (0.0–7.7)	4.7^b^ (0.0–7.8)	4.2^b^ (0.0–6.3)	5.5^a,b^ (0.0–7.3)	0.0002
Fusobacteria	7.3^a,b^ (5.5–8.8)	6.9^a,c^ (3.9–8.6)	8.2^b^ (6.0–10.3)	6.4^c^ (4.7–6.7)	7.1^a,b,c^ (3.0–7.9)	0.0014
Ruminococcaceae	7.6^a^ (2.4–8.9)	5.7^b^ (2.7–7.1)	5.6^b^ (0.0–7.5)	7.3^a,b^ (0.0–8.6)	7.9^a^ (6.5–8.6)	<0.0001
Turicibacter	2.9^a^ (0.0–7.7)	0.0^b^ (0.0–4.8)	0.0^b^ (0.0–0.0)	1.5^a,b^ (0.0–6.6)	3.8^a^ (0.0–5.9)	0.0003
Universal	12.0 (10.9–13.2)	10.8 (8.3–12.7)	11.6 (10.7–12.4)	12.0 (9.7–12.2)	12.3 (8.2–12.8)	0.0935

* Medians not sharing a common superscript are significantly different (p<0.05 based on a Dunn’s multiple comparisons test).

NHD = acute non-hemorrhagic diarrhea; AHD = acute hemorrhagic diarrhea; IBD = inflammatory bowel disease

**Figure 2:**
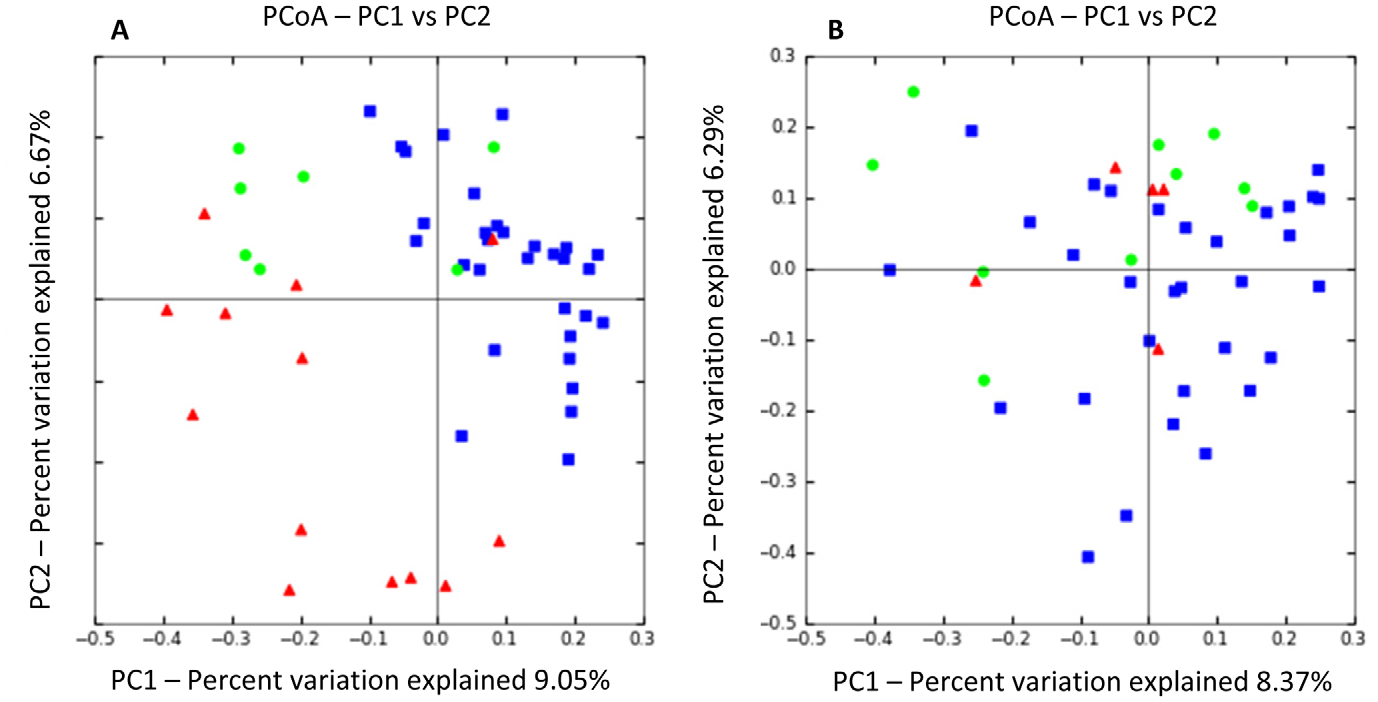
Principal Coordinates Analysis (PCoA) of unweighted UniFrac distances of 16 S rRNA genes. (A) Analysis for healthy dogs (blue), dogs with acute non-hemorrhagic diarrhea (NHD; green), and dogs with acute hemorrhagic diarrhea (AHD; red). These results indicate that fecal microbial communities differ in dogs with acute forms of diarrhea compared to healthy control dogs. Statistical analysis revealed a significant separation between samples obtained from NHD and AHD (ANOSIM; p = 0.004) and both groups were also significantly different from the healthy dogs (ANOSIM; NHD vs. healthy dogs, p = 0.003; AHD vs. healthy dogs, p = 0.001). (B) Analysis for healthy dogs (blue), dogs with active IBD (red), and dogs with therapeutically controlled IBD (green). In contrast to the dogs with acute diarrhea, fecal communities in dogs with chronic forms of diarrhea (active idiopathic IBD) were not significantly different from healthy dogs in this study.

PCoA plots (Fig. 2) were constructed to compare the individual groups of dogs, and showed notable differences between healthy dogs and dogs with acute GI disorders (ANOSIM; NHD vs. healthy dogs, p = 0.003; AHD vs. healthy dogs, p = 0.001). Furthermore, both acute disease groups differed significantly from each other (Fig. 2) (ANOSIM; NHD vs. AHD, p = 0.004).

In contrast, neither the A_IBD nor the S_IBD group were significantly different from the healthy group or different from each other based on PCoA plots (ANOSIM; A_IBD vs. S­_IBD, p = 0.91; A_IBD vs. controls, p = 0.75; S_IBD vs. controls, p = 0.07). However, sequence analysis and qPCR results revealed that *Faecalibacterium* spp. and Fusobacteria were significantly lower in dogs with clinically active IBD when compared to healthy dogs (see below).

#### Dogs with acute hemorrhagic diarrhea (AHD)

**Figure 3:**
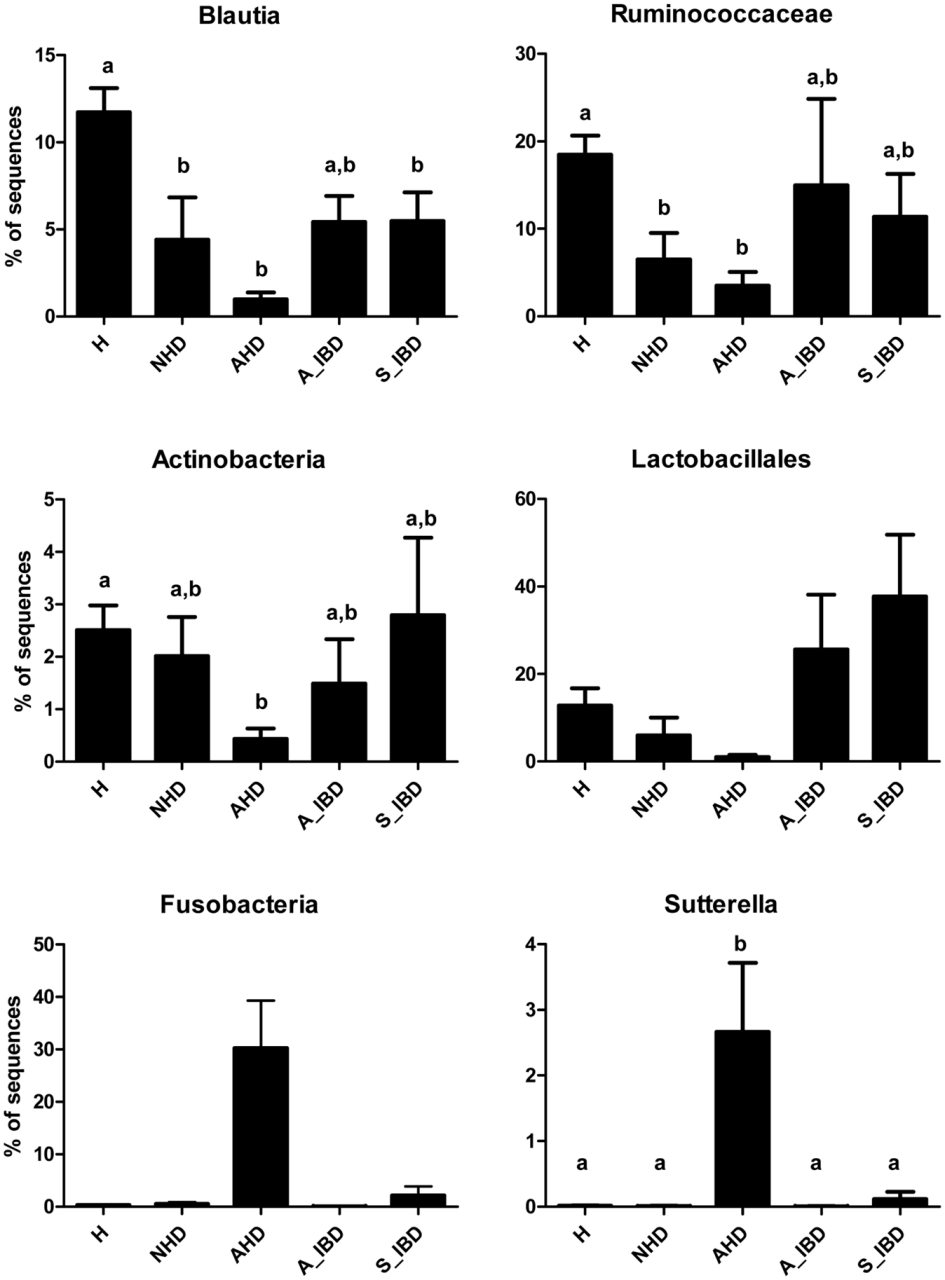
Results of sequence analysis for selected bacterial groups. H = healthy, NHD = acute non-hemorrhagic diarrhea, AHD = acute hemorrhagic diarrhea, A_IBD = active IBD, S_IBD = therapeutically controlled, clinically insignificant IBD. Columns not sharing a common superscript are significantly different (P<0.05).

Based on PCoA plots, dogs with AHD had profound microbiome changes. Several bacterial groups were altered in dogs with AHD compared to the healthy dogs, but also to dogs with other forms of GI disease (Table 3). Increases in proportions were observed for the genus *Sutterella* (class β-Proteobacteria) and *Clostridium perfringens­*-like sequences (Table 3, Fig. 3). The phylum Fusobacteria was also increased in dogs with AHD (Fig. 3), but this difference did not reach significant differences when p-values were adjusted for multiple comparisons (p = 0.08). Decreases in proportions were observed for Actinobacteria (i.e., Coriobacteriaceaea) and several members within the Firmicutes, most notably Ruminococcaceae, *Blautia* spp. (Lachnospiraceae), and *Turicibacter* spp. (Erysipelotrichaceae) (Table 3, Fig. 3).

**Figure 4:**
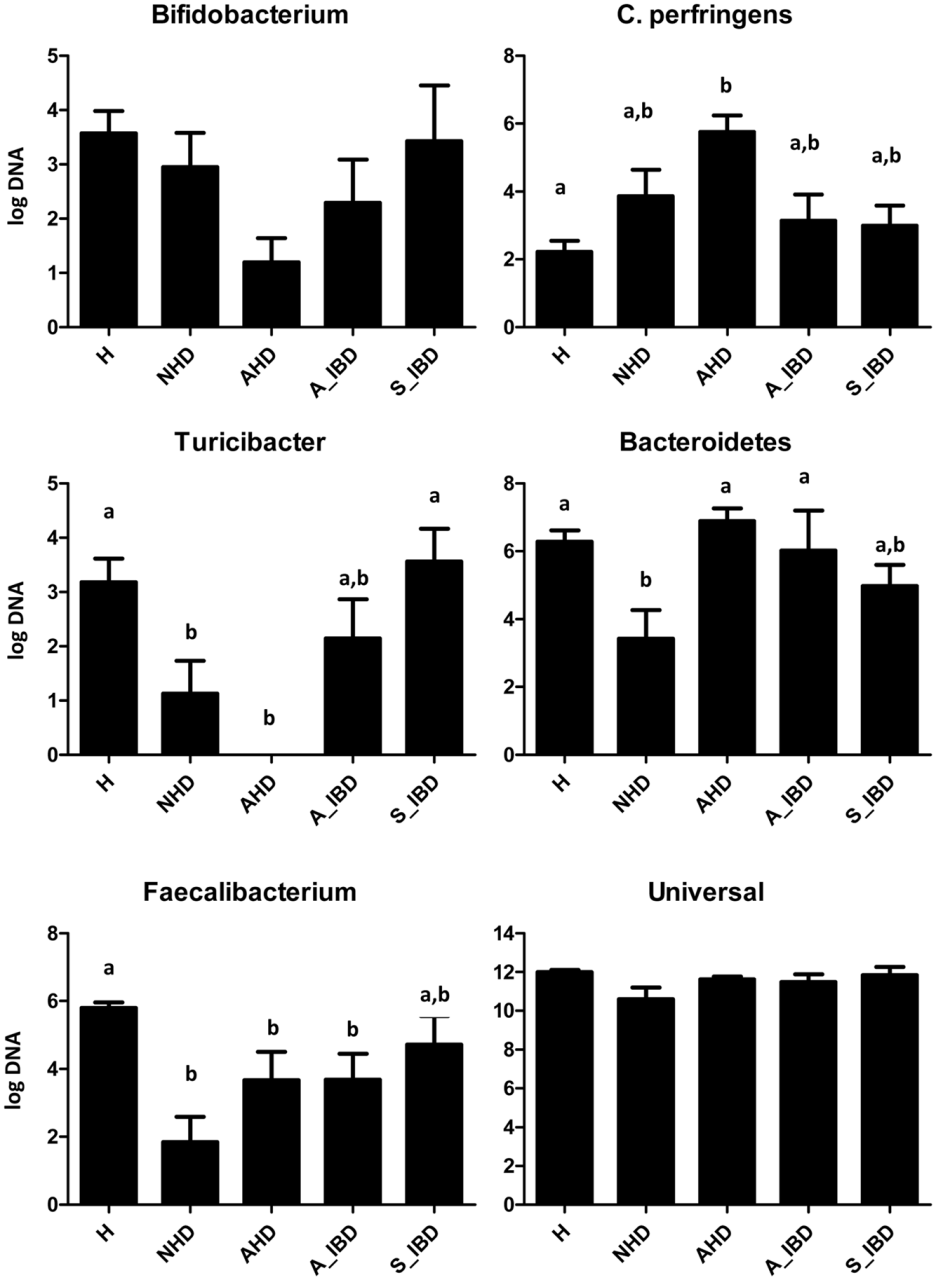
Results of quantitative PCR assays for selected bacterial groups. H = healthy, NHD = acute non-hemorrhagic diarrhea, AHD = acute hemorrhagic diarrhea, A_IBD = active IBD, S_IBD = therapeutically controlled, clinically insignificant IBD. Columns not sharing a common superscript are significantly different (P<0.05).

Results of qPCR assays confirmed significant decreases in Blautia, Ruminococcaceae including *Faecalibacterium*, and *Turicibacter* spp. (Fig. 4). *Clostridium perfringens* was significantly increased only in the dogs with AHD when compared to healthy dogs.

#### Dogs with acute non-hemorrhagic diarrhea (NHD)

PCoA plots also revealed shifts in the fecal microbiome of dogs with NHD (ANOSIM; NHD vs. healthy dogs, p = 0.003). While several bacterial taxa showed similar trends as observed for dogs with AHD, only few groups reached significance (Table 3). Sequence analysis revealed decreased proportions of *Blautia* spp. and Ruminococcaceae. Analysis by qPCR confirmed these decreases and also revealed decreases in *Turicibacter* spp., *Faecalibacterium* spp., and Bacteroidetes (Table 4).

#### Dogs with idiopathic IBD

**Figure 5:**
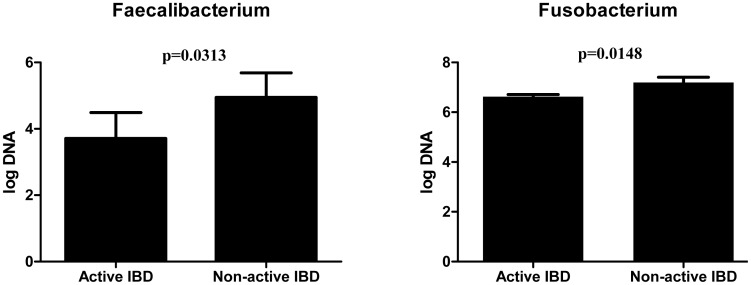
*Faecalibacterium* spp. and the phylum Fusobacteria in active and non-active IBD. Using qPCR, paired fecal samples were analyzed from dogs (n = 8) at time periods of active and clinically insignificant IBD as scored by a clinical IBD disease activity index (CIBDAI). The time period between the collections of repeated samples ranged from 2–8 months (median 5.5 months). None of the other bacterial groups evaluated by qPCR, including total bacteria, revealed significant differences between the paired time periods.

PCoA plots did not indicate a significant separation between dogs with idiopathic IBD and control dogs (Fig. 2.). However, trends were observed for decreases in proportions of *Faecalibacterium* spp. and *Turicibacter* spp. (p = 0.06) in active IBD when compared to the healthy dogs, and the proportions of these groups tended to be similar to control dogs in the samples of dogs with controlled IBD. One reason for lack of significance in the sequencing results was most likely due to the low percentage of sequencing tags for *Faecalibacterium*, with medians of 0.1% in healthy dogs and 0.0% in diseased animals. However, *Faecalibacterium* was detectable in 23/32 healthy dogs, 6/10 dogs with controlled IBD, but only 1/5 with active IBD (p = 0.04 vs. healthy dogs). Results of qPCR analysis for *Faecalibacterium* spp. confirmed the trend observed for the pyrosequencing results and showed a significant decrease in dogs with active IBD, but no significant difference between the healthy dogs and dogs with clinically insignificant IBD. Furthermore, when paired samples were analyzed from dogs (n = 8) at time periods of active and clinically insignificant IBD, the abundance of *Faecalibacterium* was significantly higher in samples collected during time periods of clinically insignificant IBD (Fig. 5, p = 0.0313).

Based on qPCR analysis, also Fusobacteria were significantly decreased in dogs with active IBD compared to the healthy dogs. Fusobacteria were also significantly increased in samples collected during time periods of clinically insignificant IBD compared to those during active disease when paired samples were analyzed (Fig. 5, p = 0.0148).

None of the other bacterial groups evaluated by qPCR, including total bacteria, revealed significant differences between healthy dogs and those with active IBD, or between time periods of active disease vs. time periods of clinically insignificant IBD.

## Discussion

This study evaluated the fecal microbiome of healthy dogs and dogs with acute and chronic GI disorders. Significant differences were observed in microbiome composition among the various disease groups. Dogs with acute diarrhea showed the most profound alterations in their microbiome. *Faecalibacterium* spp. and the phylum Fusobacteria were decreased in active IBD, but not significantly different during time periods of clinically insignificant IBD. Rarefaction curves (Fig. 1.) and alpha diversity measures (Table 1) suggested a trend for lower species richness and microbial diversity in the diseased groups. However, statistical differences (e.g., p = 0.053 for the Chao1) were not identified, most likely due to the large inter-animal variation and the relative small number of animals analyzed.

**Figure 6:**
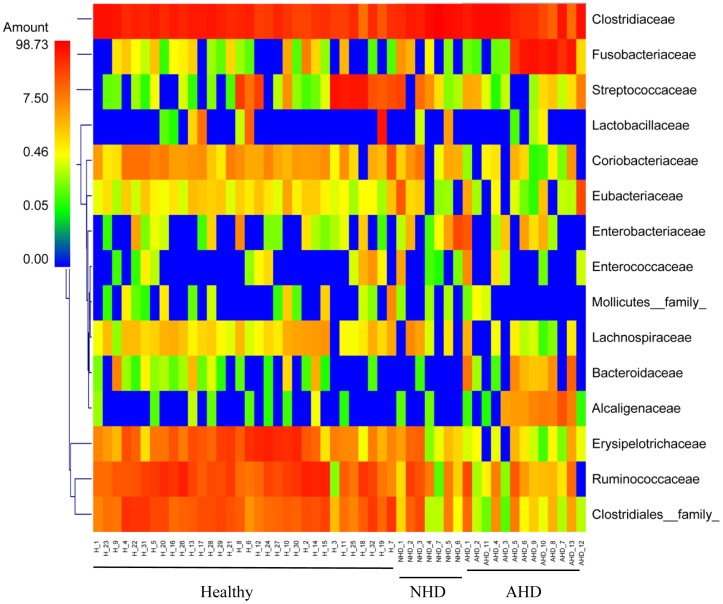
Heatmap illustrating the relative abundance of predominant bacterial families in fecal samples of healthy dogs and dogs with acute diarrhea based on 454-pyrosequencing. Samples from healthy dogs (H), dogs with acute non-hemorrhagic diarrhea (NHD), and dogs with acute hemorrhagic diarrhea (AHD) are shown. The heatmap represents the relative percentage of each family within each sample.

Various pathogens, but also other causes such as hypersensitivities, have been associated with causing acute diarrhea [27], [28]. In this study only a partial evaluation for bacterial and parasitic enteropathogens was conducted. Potential pathogens identified were *E. coli*, *Isospora*, *Giardia/Cryptosporidium,* enterotoxigenic *C. perfringens,* and toxigenic *C. difficile* (Table S2), but no clear evidence for an association between specific pathogens and acute diarrhea was identified. Patients were, therefore, classified based on clinical signs (i.e., AHD and NHD) rather than the presence of specific pathogens. The results of this study are in general agreement with previous molecular studies examining the fecal microbiota of dogs with diarrhea. In one study, dogs with acute episodes of diarrhea had an increased abundance of *C. perfringens* and *Enterococcus* spp., and a decrease in *Bacteroides* spp. [15]. Decreased proportions of Bacteroidetes were also observed in 9 dogs with unspecified diarrhea when compared to 9 healthy dogs [14]. Similarly, this study observed significant decreases in Bacteroidetes in the dogs with acute non-hemorrhagic diarrhea, but interestingly not in dogs with AHD. We also observed significant increases in *Clostridium* spp. in dogs with NHD and increases in *C. perfringens* in dogs with AHD (Fig. 4). Additionally, dogs with AHD showed increases in Fusobacteria and the genus *Sutterella* (family Alcaligenaceae). We also observed that dogs with acute diarrhea displayed significant decreases in prominent members of the intestinal microbiota, such as Erysipelotrichaceae (i.e., genus *Turicibacter*), Ruminococcaceae (i.e., *Ruminocococcus*, *Faecalibacterium)* and Lachnospiraceae (i.e., *Blautia*) (Fig. 6). Some of these groups are believed to be important producers of short-chain fatty acids (SCFA), which are important for intestinal health. For example, butyrate protects against colitis by inducing apoptosis in cells with DNA damage, while acetate beneficially modulates intestinal permeability [29], [30]. The members of the intestinal microbiota produce various other metabolites (e.g., indole) that have direct immunomodulatory properties [30], [31]. Future studies are warranted to evaluate microbiome function (e.g., microbial derived metabolites) in dogs with acute diarrhea.

Idiopathic IBD is a common chronic GI disorder of dogs. As in humans, an interplay between the intestinal microbiota together with an underlying genetic susceptibility of the host and dietary and environmental factors, are implicated in the development of disease [7], [32]. While recent studies have reported a dysbiosis in duodenal samples of dogs with IBD, limited data are available describing the fecal microbiota in these dogs [7]–[10]. In this study, *Faecalibacterium* spp. and Fusobacteria were significantly decreased in dogs with active IBD. Furthermore, when paired fecal samples were analyzed, the abundances of *Faecalibacterium* spp. and Fusobacteria were significantly decreased in samples collected during episodes of clinically active disease vs. periods of clinically insignificant IBD (Fig. 5). In contrast to previous findings in duodenal biopsies of dogs with IBD [7], we did not observe significant differences in members of Proteobacteria between healthy dogs and dogs with IBD. Proteobacteria were only significantly higher in dogs with acute diarrhea.

*Faecalibacterium* spp. were found decreased in dogs with acute diarrhea and active IBD. *Faecalibacterium prausnitzii* has garnered attention as it is often observed to be decreased in humans with IBD [33]. Furthermore it has been shown to secrete anti-inflammatory peptides in *in-vitro* studies [34]. Recent studies suggest that *Faecalibacterium* spp. are prominent members of the canine gut microbiota, as FISH analysis of fecal samples of healthy dogs estimated the abundance of the *Faecalibacterium*–*Subdoligranulum* group as a median 16% of total bacterial counts [21]. It has been suggested that dogs may harbor *Faecalibacterium* spp. other than *F. prausnitzii*, as an initial phylogenetic assessment of a near-full-length 16S rRNA gene clone obtained from the canine colon clustered distinct from a human strain of *F. prausnitzii* (AJ270469) [35]. Therefore, the phylogenetic classification of *Faecalibacterium* spp and their functional properties in the canine intestine deserve further research.

As a limitation to this study, we evaluated only a small number of animals in the various disease groups. We initially evaluated only a subset of animals by 454-pyrosequencing, as these samples were initially available at the time of sequence analysis. Therefore, due to the small sample size we may have missed some bacterial groups that are altered in certain groups of diseased dogs. Our study population was also not homogenous, as some of the dogs lived in different countries. Geographical differences in fecal microbiota have not been well examined in dogs. In this study we evaluated samples from control dogs and diseased dogs from Sweden, and also control and diseased dogs (NHD) from Texas. We did not have matching controls from Germany, the site where samples from dogs with hemorrhagic diarrhea were collected, as these were part of an unrelated study. For initial evaluation for potential differences in the canine fecal microbiome based on country and other variables, we performed clustering based on the Unifrac distance metric on all healthy dogs in this study and did not observe clustering based on country of origin (USA vs. Sweden), weight or gender (data not shown). We also performed a separate Unifrac analysis on all dogs from Sweden (healthy vs. active IBD vs. controlled IBD), and similar to the analysis that contained all dogs regardless of country of origin, we did not observe clustering between healthy dogs and dogs with IBD, but sequence and qPCR analysis revealed similar results as observed when all dogs were included in the analysis. These results would also suggest that dog microbiota could potentially be classified into specific enterotypes [36], [37]. In humans, enterotypes are characterized as compositional categories of organisms, most notably *Bacteroides*, *Prevotella*, or *Ruminococcus*, respectively, which are typically independent of nationality, gender, age, or short-term dietary interventions [36], [37]. Definition of specific microbial community enterotypes may open up new therapeutic approaches to companion animal health, by designing or prescribing appropriate diets for specific disease phenotypes.

In conclusion, we observed differences in the fecal microbiome composition between dogs with acute and chronic diarrhea compared to healthy dogs. These changes were more profound in dogs with acute diarrhea, and were not identified in dogs with therapeutically controlled IBD. These results form a roadmap for additional studies focused on a more defined population of diseased dogs.

## Supporting Information

Table S1 **Control dogs enrolled into the study.**
(PDF)

Table S2 **Dogs with acute hemorrhagic diarrhea (AHD) and acute non-hemorrhagic diarrhea (NHD).**
(PDF)

Table S3 **Dogs with active IBD and with therapeutically controlled IBD.**
(PDF)

Methods S1 **Linear discriminant analysis.**
(PDF)
