# Supplementary material for: The Fecal Microbiome in Dogs with Acute Diarrhea and Idiopathic Inflammatory Bowel Disease
Source: PLoS One. 2012 Dec 26;7(12):e51907. doi: 10.1371/journal.pone.0051907 (PMC3530590; doi:10.1371/journal.pone.0051907)
Supplement: Table S1 — Control dogs enrolled into the study. (PDF) [file pone.0051907.s001.pdf]

Table S1. Control dogs enrolled into the study

ID	Age (yrs)	Breed	Sex	Weight (lbs)	Antibiotic history	Diet
H1	0.3	Border Terrier	m	11.0	none	Eukanuba lamb and rice
H2	10.8	Mixed breed	m	72.6	none	Hill's Science Plan
H3	9.7	Samoyed	m	70.4	Chloramphenicol opthalmic (3.5 months before sampling)	Hill's Science Plan lamb and rice Senior
H4	2.5	Mixed breed	f	9.9	none	Hill´s Science Plan Mini Adult (chicken)
H5	1.8	Border Terrier	f	12.8	Polymyxin B sulfate otic (4 months before sampling)	Eukanuba lamb and rice
H6	10.9	Mixed breed	m	70.4	none	Hill's Performance
H7	14.9	Mixed breed	mn	12.3	none	Hill's Prescription i/d, k/d and cottage cheese
H8	5.5	Tervuren	f	48.4	Pivampicillin (3 months before sampling)	Hill´s Science Plan Adult (chicken)
H9	10.2	Mixed breed	fs	63.0	none	Purina OM
H10	0.8	Mixed breed	m	10.2	none	IAMS puppy
H11	3.7	Blue Heeler	fs	59.5	none	Hill's science diet weight loss
H12	1.9	Labrador Retriever	f	55.5	none	unknown
H13	3.5	Miniature Dachshund	mn	11.5	none	Hill's Science diet adult
H14	2.8	Rhodesian Ridgeback mix	mn	69.0	none	Hill's Science Plan diet large breed
H15	3.7	Jack Russel mix	fs	10.0	none	IAMS FP
H16	0.9	Pembroke Welsh Corgi	fs	24.8	none	Purina Pro Plan
H17	0.7	Mixed breed	mn	35.0	none	Purina premium puppy
H18	5.0	Boxer	mn	75.0	none	Hill's Science diet advanced
H19	7.9	Chesapeake Bay Retriever	fs	64.0	none	Purina JM
H20	6.9	Labrador Retriever	mn	81.5	none	Purina EN
H21	4.2	Shih-tzu	fs	15.3	none	unknown
H22	4.0	English Bulldog	fs	45.5	none	Dick van Potton (Duck/Potato)
H23	15.0	Chihuahua	fs	5.8	none	unknown
H24	2.0	Golden Retriever	mn	77.1	none	Pedigree
H25	7.8	Mixed breed	mn	66.9	none	unknown
H26	4.0	Dachshund	fs	13.2	none	Hill’s Prescription s/d adult
H27	9.6	Mixed breed	fs	70.7	none	Science Duck & Potato Light
H28	10.0	Mixed breed	mn	61.7	none	Royal Canine
H29	3.5	Miniature Schnauzer	mn	21.0	none	Hill’s Prescription s/d adult
H30	5.2	Terrier Mix	mn	61.0	none	unknown
H31	5.9	Brussels Griffon	mn	14.0	none	unknown
H32	9.2	Beagle	mn	27.2	none	Science Diet Light

m=male intact; f=female intact; mn=male neutered; fs=female spayed
