# Supplementary material for: The Fecal Microbiome in Dogs with Acute Diarrhea and Idiopathic Inflammatory Bowel Disease
Source: PLoS One. 2012 Dec 26;7(12):e51907. doi: 10.1371/journal.pone.0051907 (PMC3530590; doi:10.1371/journal.pone.0051907)
Supplement: Table S2 — Dogs with acute hemorrhagic diarrhea (AHD) and acute non-hemorrhagic diarrhea (NHD). (PDF) [file pone.0051907.s002.pdf]

Table S2. Dogs with acute hemorrhagic diarrhea (AHD) and acute non-hemorrhagic diarrhea (NHD)

ID	Age (yrs)	Breed	Sex	Weight (lbs)	Antibiotic history	Disease	CPE (ELISA)	*cpe* (PCR)	*C. difficile* toxin A/B (ELISA)	gene for *C. difficile* toxin B (PCR)	culture/fecal cytology	Giardia/parasites
HD1	8.0	Yorkshire Terrier	m	4.0	none	AHD	-	+	+	+	-	n/a
HD2	3.0	Mixed breed	fs	26.0	none	AHD	+	+	-	-	n/a	n/a
HD3	12.0	Mixed breed	m	17.6	none	AHD	-	-	-	-	-	-
HD4	7.0	Jack Russell Terrier	fs	19.8	none	AHD	-	+	-	-	-	Giardia
HD5	5.0	Mixed breed	m	68.3	none	AHD	+	+	-	-	hem. E. coli	-
HD6	13.0	Viszla	mn	61.7	none	AHD	-	+	-	-	hem. E. coli	-
HD7	3.0	Mixed breed	fs	11.1	none	AHD	+	+	-	-	-	n/a
HD8	16.0	Poodle	fs	19.4	none	AHD	-	+	-	-	hem. E. coli	-
HD9	7.0	Labrador mix	fs	62.8	none	AHD	-	+	-	-	-	-
HD10	2.0	Beagle	m	25.1	none	AHD	-	-	-	-	-	Giardia pos
HD11	4.0	Jack Russell Terrier	fs	13.8	none	AHD	-	+	-	-	-	n/a
HD12	3.0	Jack Russell Terrier	fs	9.5	none	AHD	-	+	-	-	hem. E. coli	-
HD13	4.0	Mixed breed	m	57.3	none	AHD	-	+	-	-	-	-
D1	1.5	Labrador Retriever	fs	61.7	none	NHD	-	+	-	-	+++ EFR	-
D2	11.0	Mixed Breed	fs	31.9	none	NHD	-	-	-	-	naf	-
D3	15.0	Cocker Spaniel	mn	35.3	none	NHD	-	-	-	-	naf	-
D4	7.0	Chihuahua	fs	5.5	none	NHD	+	n/a	n/a	n/a	+++ EFR	-
D5	2.3	King Charles	fs	13.2	none	NHD	+	+	-	-	+EFR	-
D6	14.0	Golden Retriever	fs	54.5	none	NHD	-	+	-	-	+EFR	-
D7	1.0	Labrador mix	mn	39.6	none	NHD	-	+	-	-	+++ EFR	-
D8	3.5	Weimaraner	m	62.0	none	NHD	-	-	-	-	+EFR	C/G
D9	8.5	Labrador Retriever	fs	60.0	none	NHD	-	+	-	-	+EFR	-
D12	0.5	Border Terrier	m	16.0	none	NHD	-	-	-	-	naf	Isospora
D14	1.8	German Shepherd Dog	fs	70.0	none	NHD	-	-	-	-	naf	-
D16	9.5	Rottweiler mix	fs	75.0	none	NHD	+	-	-	-	naf	-

m=male intact; f=female intact; mn=male neutered; fs=female spayed

EFR=endospore forming rods; C/G=positive for Cryptosporidium and Giardia on PCR

CPE=*C. perfringens* enterotoxin by ELISA; *cpe*=gene for *C. perfringens* enterotoxin (PCR)

naf=no abnormalities found; n/a=not evaluated
