# Supplementary material for: The Fecal Microbiome in Dogs with Acute Diarrhea and Idiopathic Inflammatory Bowel Disease
Source: PLoS One. 2012 Dec 26;7(12):e51907. doi: 10.1371/journal.pone.0051907 (PMC3530590; doi:10.1371/journal.pone.0051907)
Supplement: Table S3 — Dogs with active IBD and with therapeutically controlled IBD. (PDF) [file pone.0051907.s003.pdf]

Table S3. Dogs with active IBD and with therapeutically controlled IBD

Active IBD	Age (yrs)	Breed	Sex	Weight (lbs)	CIBDAI	Histopathology	Therapy/Diet at time of sample collection
A_IBD_1	6.5	Golden Retriever	n/a	74.8	7	Lymphocytic-plasmacytic gastritis, eosinophilic enteritis;	Hill's advanced chicken and rice
A_IBD_3	7.6	Bichon Frisé	f	15.2	5	Lymphocytic-plasmacytic gastritis/colitis	Hill's Prescription i/d
A_IBD_4	4.4	Bullmastiff	fs	94.2	5	Moderate chronic active gastritis, duodenitis, colitis, mixed cell-type	Fresh boiled rumen + cereals
A_IBD_5	3.9	Great Dane	m	130	7	Moderate mixed cell-type (lymphocytes, plasma cells, macrophages) gastritis, duodenitis, and colitis	James Wellbeloved Lamb and rice
A_IBD_6	3.5	Shetland Sheepdog	m	18.5	8	Moderate to severe follicular gastritis, mild lymphocytic-plasmacytic, moderate Lymphocytic-plasmacytic chronic colitis	Eukanuba Jogging Agility
A_IBD_7	0.8	Boxer	m	55	9	Moderate eosinophilic chronic active colitis, moderate chronic active gastritis	James Wellbeloved Duck and rice
A_IBD_8	0.6	Papillon	mn	9	6	Moderate lymphocytic-plasmacytic enteritis with villous atrophy, moderate lymphocytic-plasmacytic colitis	Royal Canin Urinary
A_IBD_9	2.4	Miniature Poodle	m	12.3	8	Moderate lymphocytic-plasmacytic gastritis with fibrosis, normal duodenum, mild lymphocytic-plasmacytic colitis	Hill´s Science Plan Adult lamb and rice
A_IBD_10	2	Rottweiler	fs	67.1	7	Mild eosinophilic gastritis and enteritis, mild lymphocytic-plasmacytic colitis	Hill´s Prescription i/d

Controlled IBD	Age (yrs)	Breed	Sex	Weight (lbs)	CIBDAI	Histopathology	Therapy/Diet at time of sample collection
S-IBD_1	5.8	Mixed breed	m	59.4	2	Chronic moderate lymphocytic-plasmacytic colitis with polyps, mild superficial chronic gastritis	budosenide, azathioprine, olsalazine; Hill's Prescription d/d salmon and rice
S-IBD_2	8.7	Shetland Sheepdog	f	18.5	1	Moderate to severe diffuse lymphocytic-plasmacytic gastritis with fibrosis (mild- severe in different locations), moderate lymphocytic-plasmacytic enteritis, mild to moderate chronic mixed cell-type colitis	methylprednisolone, psyllium; RC Sensitivity Control
S-IBD_3	4.2	Norwegian Elkhound	m	57.2	1	Moderate chronic mixed cell-type colitis with prominent fibrosis, mild superficial chronic mixed cell-type gastritis and enteritis	prednisolone; Hill's Prescription d/d duck and rice
S-IBD_4	8	Parson Russell Terrier	m	19.1	1	Moderate chronic active mixed cell-type gastritis and enteritis, mild colitis	methylprednisolone, azathioprine, psyllium; Hill's d/d duck and rice
S-IBD_5	3.8	Hungarian Vizsla	m	55	2	Moderate lymphocytic-plasmacytic colitis with multiple erosions and moderate fibrosis, moderate chronic mixed cell-type gastritis	methylprednisolone, psyllium; RC Sensitivity Control
S-IBD_6	5.7	Weimaraner	fs	70.4	1	Chronic lymphocytic-plasmacytic enteritis and colitis	budosenide, azathioprine; RC Hypoallergenic
S-IBD_7	3.7	Mixed breed	m	74.4	2	Moderate chronic active mixed cell-type follicular gastritis, moderate chronic mixed cell-type enteritis and colitis	prednisolone; Bento Kronen lamb and rice
S-IBD_8	4	Mixed breed	mn	19.1	1	Moderate chronic active mixed cell-type gastritis and colitis, mild enteritis. Mild-moderate patchy fibrosis of stomach and colon	prednisolone, ProSoluble (probiotics); Hill's Science Plan lamb and rice
S-IBD_9	6.5	Bernese Mountain Dog	m	91.7	2	Mild chronic lymphocytic-plasmacytic gastritis and colitis	methylprednisolone, azathioprine, olsalazine, metoclopramide, psyllium; Hill's Z/D
S-IBD_10	8.6	Lhasa Apso	f	18.7	2	Moderate atrophic gastritis, eosinophilic enteritis and colitis	prednisolone, cyclosporine, olsalazine; RC Hypoallergenic

m=male intact; f=female intact; mn=male neutered; fs=female spayed; IBD=inflammatory bowel disease; CIBDAI=canine IBD activity index
