# Supplementary material for: The Fecal Microbiome in Dogs with Acute Diarrhea and Idiopathic Inflammatory Bowel Disease
Source: PLoS One. 2012 Dec 26;7(12):e51907. doi: 10.1371/journal.pone.0051907 (PMC3530590; doi:10.1371/journal.pone.0051907)
Supplement: Methods S1 — Linear discriminant analysis. (PDF) [file pone.0051907.s004.pdf]

Methods S1. Linear discriminant analysis

Materials and methods

Using the OTUs as features for the classification, the single, two or three feature LDA classifiers were constructed and ranked based on their error estimates and the top performing ones were identified to discriminate between healthy dogs and dogs with gastrointestinal disease (independent of diarrhea type). Different phenotypes were assessed at the levels of various operational taxonomic units/features (OTU's) with the following respective levels: phylum, class, order, family, genus, and species. To estimate the improvements of the classification performance, we used the following error-related quantities: $\boldsymbol{\varepsilon}_{\mathbf{bolstered}}$ and $\boldsymbol{\Delta\varepsilon}_{\mathbf{bolstered}}$ [1]. $\boldsymbol{\varepsilon}_{\mathbf{bolstered}}$ denotes the bolstered re-substitution error for the LDA classifier for the respective feature set of size *n n (n = 1,2,3)*, and $\boldsymbol{\Delta\varepsilon}_{\mathbf{bolstered}}$ denotes the decrease in error with respect to the highest ranked of its subsets of features (in the list of features of size *n-1,* with n *= 2, 3)*. The feature sets were ranked based on the value of $\boldsymbol{\varepsilon}_{\mathbf{bolstered}}$.

Results

The best single, two- and three – feature LDA classifiers that discriminate between healthy dogs and dogs with diarrhea were identified.

It is believed that multivariate feature sets are better discriminators when the phenotype is complex [2]. The classification methodology shares some similarities with PCA-based analyses of data. However, the important distinction lays in the ability of the classification approach to provide means to quantify the degree of separation between the phenotypes in question. The relatively high error estimates

for the LDA classifications analyses reflect the apparent heterogeneity of the data set, and point out to the need for more focused experimental design.

In order to illustrate the performance of the LDA classification, we consider an example where the goal is to discriminate the samples from the group of healthy animals from the rest of the samples at the genus level (table 1). At that level of bacterial OTUs, our results show that *Peptococcus* is the top performing one-feature classifier with an estimated error ($\varepsilon_{\text{bolstered}}$) of ~19%. However, all of the top single feature classifiers with the exception of Blautia, were based on OTUs that were present in very few of the samples in this study. Thus, the error estimates for the single feature LDA classifiers are not reliable. It is interesting to observe that *Turicibacter* does not appear in the top five one-feature classifiers; however, when combined with *Blautia* they form the best two-feature classifier with an estimated error of ~22%. For the majority of three-feature classifiers, *Blautia* and *Turicibacter* combination is present with only the third feature being variable across the top five cases. Improvement in the classification accuracy by using *Blautia*, *Turicibacter*, and *Faecalibacterium* as a triplet classifier was around 2% ($\Delta\varepsilon_{\text{bolstered}}$) relative to its highest ranked subset of features (*Blautia* and *Turicibacter*). The separation of the healthy group from the rest of the dogs at genus level by the respective plane based on that triplet classifier is presented in Figure 1. The apparent heterogeneity in the group of healthy animals can be clearly seen, and that contributes to the relatively high estimated error rates.

Table 1. **Linear Discriminant Analysis (LDA) of healthy dogs versus the other disease groups at genus level**. The top single, pair-wise, and triplet-wise classifiers are shown. $\varepsilon_{\text{bolstered}}$ denotes bolstered re-substitution error for the respective classifiers; the classifiers are ranked according to that error measurement. $\Delta\varepsilon_{\text{bolstered}}$

denotes the decrease in the error for each feature set relative to its highest ranked subset of features.

1 feature	2 feature	3 feature	$\varepsilon_{bolstered}$	$\Delta \varepsilon_{bolstered}$
**Peptococcus**			0.1982	
**Pasteurellaceae (genus)**			0.2508	
**Alcaligenaceae (genus)**			0.2584	
**Porphyromonas**			0.2664	
Blautia			0.2697	
**Peptostreptococcus**			0.2718	
Blautia	Turicibacter		0.2177	0.052
Blautia	Clostridiales (genus)		0.2306	0.0391
Sutterella	**Olsenella**		0.2319	0.0504
Sutterella	**Actinobacillus**		0.2326	0.0497
Sutterella	**Moraxellaceae (genus)**		0.233	0.0493
Blautia	Clostridiales (genus)	Turicibacter	0.1913	0.0264
Blautia	Eubacterium	Turicibacter	0.1978	0.0199
Blautia	Faecalibacterium	Turicibacter	0.1987	0.019
Blautia	**Coprobacillus**	Turicibacter	0.205	0.0127
Blautia	Clostridiales (genus)	**Coprobacillus**	0.2099	0.0207

In bold: these groups were present only in individual dogs

Figure 1. Linear Discriminant Analysis (LDA) classification for the case healthy, X versus all dog groups with diarrhea, Y at the genus level.
Blautia, *Turicibacter,* and *Faecalibacterium* provided one of the top five performing feature sets of size 3. The three LDA plane discriminates between X (circles) and Y (triangles). Axes represent population abundance (%) of the respective OTUs.

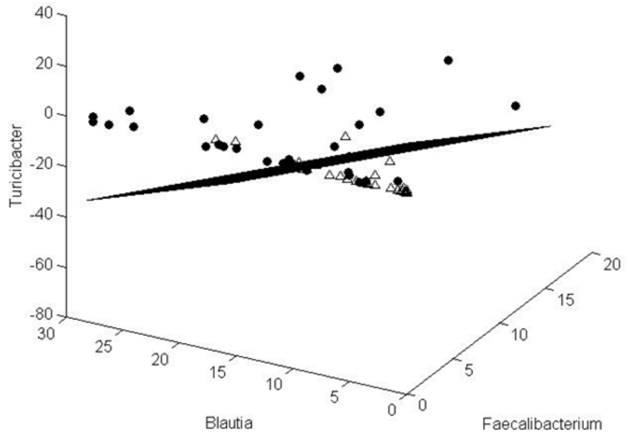

References

1. Sima C, Braga-Neto UM, Dougherty ER (2011) High-dimensional bolstered error estimation. Bioinformatics 27: 3056-3064.
2. Chapkin RS, Zhao C, Ivanov I, Davidson LA, Goldsby JS, et al. (2010) Noninvasive stool-based detection of infant gastrointestinal development using gene expression profiles from exfoliated epithelial cells. Am J Physiol Gastrointest Liver Physiol 298: G582-589.
